# Outcomes of steroid-resistant nephrotic syndrome in children not treated with intensified immunosuppression

**DOI:** 10.1007/s00467-022-05762-4

**Published:** 2022-10-31

**Authors:** Agnes Trautmann, Svenja Seide, Beata S. Lipska-Ziętkiewicz, Fatih Ozaltin, Maria Szczepanska, Marta Azocar, Augustina Jankauskiene, Alexandra Zurowska, Salim Caliskan, Bassam Saeed, William Morello, Francesco Emma, Mieczyslaw Litwin, Alexey Tsygin, Svitlana Fomina, Anna Wasilewska, Anette Melk, Elisa Benetti, Jutta Gellermann, Natasa Stajic, Marcin Tkaczyk, Sergey Baiko, Larisa Prikhodina, Dagmar Csaicsich, Anna Medynska, Regina Krisam, Heike Breitschwerdt, Franz Schaefer, for the PodoNet Consortium

**Affiliations:** 1 Division of Pediatric Nephrology, University Center for Pediatrics and Adolescent Medicine, Im Neuenheimer Feld 430, 69120 Heidelberg, Germany; 2 Institute of Medical Biometry, University of Heidelberg, Heidelberg, Germany; 3 Rare Diseases Centre and Clinical Genetics Unit, Department of Biology and Medical Genetics, Medical University of Gdansk, Gdansk, Poland; 4 Department of Pediatric Nephrology, Nephrogenetics Laboratory and Center for Biobanking and Genomics, Hacettepe University, Ankara, Turkey; 5 Department of Pediatrics, School of Medicine With the Division of Dentistry, Zabrze, Poland; 6 Pediatric Nephrology, Hospital Luis Calvo Mackenna-Facultad de Medicina Universidad de Chile, Santiago de, Chile; 7 Pediatric Center, Institute of Clinical Medicine, Vilnius University, Vilnius, Lithuania; 8 Department of Pediatrics, Medical University of Gdansk, Nephrology & Hypertension, Gdansk, Poland; 9 Pediatric Nephrology Department, Cerrahpaşa Medical Faculty, Istanbul University-Cerrahpasa, Istanbul, Turkey; 10 Farah Association for Child With Kidney Disease in Syria, Damascus, Syria; 11 Pediatric Nephrology, Dialysis and Transplant Unit, Fondazione IRCCS Ca’Granda, Ospedale Maggiore Policlinico, Milano, Italy; 12 Department of Pediatric Subspecialties, Nephrology and Dialysis Unit, Children’s Hospital Bambino Gesù, IRCCS, Rome, Italy; 13 Department of Pediatric Nephrology, The Children’s Memorial Health Center, Warsaw, Poland; 14 National Medical and Research Center for Children’s Health, Moscow, Russia; 15 Department of Pediatric Nephrology, State Institution “Institute of Nephrology of NAMS of Ukraine”, Kyiv, Ukraine; 16 Department of Pediatric Nephrology, University Hospital, Bialystok, Poland; 17 Department of Pediatric Kidney, Liver and Metabolic Diseases, Hannover Medical School, Hannover, Germany; 18 Pediatric Nephrology, Dialysis and Transplant Unit, Department of Women’s and Children’s Health, Padua University Hospital, Padua, Italy; 19 Clinic for Pediatric Nephrology, Charite Hospital, Berlin, Germany; 20 Department of Pediatric Nephrology, Institute of Mother and Healthcare of Serbia, Belgrade, Serbia; 21 Pediatric Nephrology Division, Polish Mothers Memorial Hospital Research Institute, Lodz, Poland; 22 National Center for Pediatric Nephrology and Renal Replacement Therapy, Belarusian State Medical University, Minsk, Belarus; 23 Division of Inherited & Acquired Kidney Diseases, Research & Clinical Institute for Pediatrics, Pirogov Russian National Research Medical University, Moscow, Russia; 24 Department of Pediatrics, Medical University Vienna, Vienna, Austria; 25 Department of Pediatric Nephrology, Wroclaw Medical University, Wroclaw, Poland

**Keywords:** Nephrotic syndrome, Steroid resistance, SRNS, Children, Antiproteinuric treatment

## Abstract

**Background:**

The aim of the current PodoNet registry analysis was to evaluate the outcome of steroid-resistant nephrotic syndrome (SRNS) in children who were not treated with intensified immunosuppression (IIS), focusing on the potential for spontaneous remission and the role of angiotensin blockade on proteinuria reduction.

**Methods:**

Ninety-five pediatric patients who did not receive any IIS were identified in the PodoNet Registry. Competing risk analyses were performed on 67 patients with nephrotic-range proteinuria at disease onset to explore the cumulative rates of complete or partial remission or progression to kidney failure, stratified by underlying etiology (genetic vs. non-genetic SRNS). In addition, Cox proportional hazard analysis was performed to identify factors predicting proteinuria remission.

**Results:**

Eighteen of 31 (58.1%) patients with non-genetic SRNS achieved complete remission without IIS, with a cumulative likelihood of 46.2% at 1 year and 57.7% at 2 years. Remission was sustained in 11 children, and only two progressed to kidney failure. In the genetic subgroup (*n* = 27), complete resolution of proteinuria occurred very rarely and was never sustained; 6 (21.7%) children progressed to kidney failure at 3 years. Almost all children (96.8%) received proteinuria-lowering renin–angiotensin–aldosterone system (RAAS) antagonist treatment. On antiproteinuric treatment, partial remission was achieved in 7 of 31 (22.6%) children with non-genetic SRNS and 9 of 27 children (33.3%) with genetic SRNS.

**Conclusion:**

Our results demonstrate that spontaneous complete remission can occur in a substantial fraction of children with non-genetic SRNS and milder clinical phenotype. RAAS blockade increases the likelihood of partial remission of proteinuria in all forms of SRNS.

**Graphical abstract:**

**Figure:**
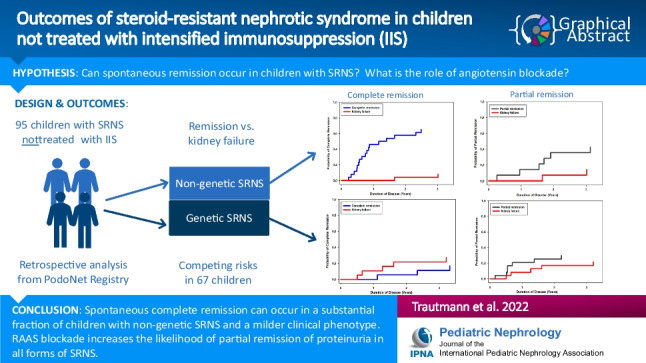
A higher resolution version of the Graphical abstract is available as Supplementary information

**Supplementary Information:**

The online version contains supplementary material available at 10.1007/s00467-022-05762-4.

## Introduction

Approximately 10–15% of children with idiopathic nephrotic syndrome demonstrate resistance to standard oral steroid therapy. The etiology of steroid resistant nephrotic syndrome (SRNS) is heterogeneous and the disease course is highly variable. Whereas 20–30% of cases can be attributed to defects in podocyte-associated genes [1–4], the etiology of the remaining 70–80% of “idiopathic” SRNS cases is still largely elusive. In children with non-genetic SRNS, calcineurin inhibitor (CNI)–based intensified immunosuppression (IIS) is the recommended first-line therapeutic approach [5]. The response to IIS depends on the underlying etiology and has been shown to be predictive of the kidney outcome [6–9]. Children with proven genetic SRNS are unlikely to respond to IIS [5, 9].

While current clinical practice guidelines recommend IIS in all patients diagnosed with SRNS—ideally after ruling out a causative genetic defect—the evidence base supporting the efficacy of CNI and other immunosuppressants from placebo-controlled RCTs is limited and mostly relies on non-placebo-controlled RCTs comparing different immunosuppressive agents and retrospective observational studies [10–19]. The usefulness and efficacy of IIS in SRNS are also well known from clinical experience within the past three decades. However, anecdotal reports have indicated that complete remission may occasionally occur in children with SRNS [20, 21]. Several clinical observations, such as the frequently observed late remission after more than 6–12 months IIS exposure and the sustained remission after IIS discontinuation in apparent treatment responders [22], point to the possibility that remission may sometimes occur independently of the medication applied. Spontaneous remission is well established in several other immune-mediated glomerulopathies and in some cases has led to risk-stratified management strategies including the option of foregoing any immunosuppressive treatment, e.g., in membranous nephropathy in adults [23].

The PodoNet Registry is the largest current database of pediatric SRNS, with more than 2000 registered children. Detailed longitudinal clinical, biochemical, genetic, and medication-related information is available in a subset of this cohort. In this work, we identified a group of SRNS patients in the PodoNet database who were never exposed to IIS and followed their disease course to document the occurrence of spontaneous remission and the impact of RAAS blockade on the course of proteinuria.

## Methods

### Patient cohort and analytical approach

**Fig. 1:**
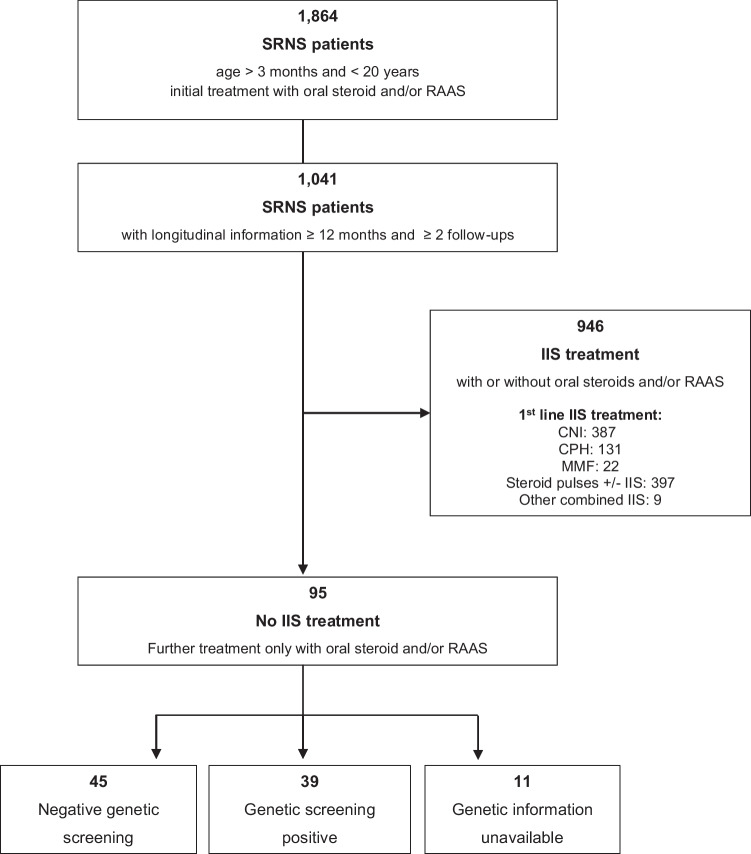
Selection of non-IIS sample from PodoNet SRNS cohort. *SRNS*, steroid-resistant nephrotic syndrome; *CNI*, calcineurin inhibitor; *CPH*, cyclophosphamide; IIS, intensified immunosuppressive treatment; *MMF*, mycophenolate mofetil; *non-IIS*, children not treated with intensified immunosuppression; *RAAS*, renin–angiotensin–aldosterone antagonist

The PodoNet registry is an international web-based clinical registry (www.podonet.org) for childhood-onset primary SRNS, congenital nephrotic syndrome (CNS), and genetic podocytopathies. The registry protocol, description, and characterization of the PodoNet cohort were previously published [2]. Among 1864 registered patients aged 4 months to 19 years at SRNS diagnosis, adequate longitudinal clinical, biochemical, and medication information was available on 1041 children (Fig. 1). Of these, 95 children were treated only with continued oral steroids and/or RAAS antagonists but did not receive any IIS at any time during follow-up (“non-IIS” cohort). The renunciation of the start of any IIS therapy and their reasons were explicitly reconfirmed by the clinical centers through individual queries.

Within this specifically selected cohort, 67 children presented nephrotic-range proteinuria at disease onset, whereas 13 showed non-nephrotic-range proteinuria. Exact initial proteinuria information at first manifestation was missing in 13 children due to incomplete retrospective reporting by the contributing clinical centers. Two children were identified with genetic SRNS following family screening before developing proteinuria.

The maximal proteinuria reduction (“best response”) and kidney function outcome were evaluated using competing risk analysis in 67 children with nephrotic-range proteinuria at disease onset, with responsiveness (complete/partial) to non-IIS treatment and kidney failure as competing events.

The diagnosis of steroid resistance and the response to non-IIS treatment were evaluated according to previously defined and published criteria based on changes in proteinuria and serum albumin [2]: complete remission was diagnosed in case of proteinuria reduction to < 100 mg/m^2^/d 24-h protein excretion, < 0.2 mg/mg protein/creatinine ratio in spot urine (UPCr) (if age < 2 years: < 0.5 mg/mg), a negative dipstick reading, or serum albumin > 30 g/l combined with dipstick trace ( +). Partial remission was defined as persistent non-nephrotic-range proteinuria with 24-h protein excretion > 100 mg/m^2^/d but < 1 g/m^2^/d, UPCr 0.2–2 mg/mg (if age < 2 years: 0.5–2 mg/mg), dipstick 1 + with serum albumin > 30 g/l or dipstick trace ( +) with serum albumin < 30 g/l. Lack of remission was defined as persistent nephrotic-range proteinuria as defined by 24-h protein excretion ≥ 1 g/m^2^/d, UPCr > 2 mg/mg, dipstick 2 + or greater, and dipstick 1 + with serum albumin ≤ 30 g/l. Kidney failure was defined by attainment of CKD stage 5 and/or start of kidney replacement therapy (KRT).

To assess RAAS antagonist exposure, dosage and duration of administration were assessed for each RAAS inhibitor compound. In addition, total relative RAAS antagonist exposure was quantitated as percentage of the maximum approved pediatric drug dosage [5] prescribed on average during the observation period. In case of combined ACEi and ARB treatment, the percentages of each drug were added up. Total relative RAAS exposure is independent of the type of RAAS medication and allows comparing the administered RAAS drug dosages across patients.

### Statistical analysis

Descriptive data are given as medians (interquartile ranges) or means (standard deviation) for continuous variables and absolute and relative frequencies for categorical variables, referring to all patients with available information on the parameter of interest. Proportions are given with normal-approximated 95% confidence intervals.

To evaluate proteinuria outcome (time to first complete or partial remission as best response), a competing risk analysis was performed treating kidney failure as a competing event. The competing risk approach was chosen since the probabilities of achieving remission or kidney failure are interrelated. Only children presenting with nephrotic-range proteinuria at disease onset were included in this analysis (*n* = 67) to avoid potential bias from inclusion of children with initial non-nephrotic range proteinuria and milder clinical phenotype. Subgroup analyses were performed according to genetic status. Sensitivity analyses were performed in patients starting from non-nephrotic proteinuria (data not shown here).

To identify factors predicting proteinuria reduction, a univariate Cox regression analysis was performed where kidney failure was treated as a censoring event, complemented by a bivariate analysis evaluating the effects of genetic status and RAAS exposure, the two variables that emerged as significant in the univariate Cox regression model. Due to the limited sample size, further evaluation such as using statistical variable selection algorithms was not possible.

## Results

### Patient characteristics

Ninety-five SRNS children in the PodoNet registry never received IIS. Thirty-nine (41 (31; 51) %) children were diagnosed with a genetic form of SRNS; in 45 (47 (37; 57) %), genetic screening was negative, and in 11 (12 (6; 17) %), children screening information was not available. Reasons not to administer IIS in children included early establishment of a genetic diagnosis (*n* = 39) and, in children with non-genetic disease, relatively mild clinical presentation (*n* = 14), late spontaneously regressing proteinuria (> 8 weeks; n = 8), familial SRNS with documented non-responsiveness in the index case (*n* = 6), disease onset between the 4th and 12th month of life (*n* = 2), presentation with impaired eGFR (*n* = 7), presence of extrarenal symptoms (*n* = 3), type of underlying histopathology (membranous nephropathy, *n* = 1), and parental non-acceptance of IIS (*n* = 2). In two children, the reason could not be ascertained retrospectively.

Patient characteristics at disease onset were similar in the non-genetic and genetic subgroups and generally indicated a relatively mild initial disease presentation in the cohort (Table 1): although 82 (74; 90) % of children presented with nephrotic-range proteinuria, hypoalbuminemia was usually mild. Up to 63 (53; 73) % of children presented without clinically relevant edema. Two children were identified with genetic disease by family screening performed because of affected siblings while they were still asymptomatic and free of proteinuria. One of these, diagnosed with homozygous *NUP205* pathogenic variants, developed nephrotic-range proteinuria at age 9 years. The other patient, compound-heterozygous for the non-neutral *NPHS2* polymorphism p.R229Q and the pathogenic variant p.E281Q, developed non-nephrotic-range proteinuria at age 2.3 years, sustained for five years before nephrotic-range proteinuria occurred.

**Table 1:** **Characteristics of 95 SRNS patients who never received intensified immunosuppressive therapy**. Data are given as N (% (normal-approximated 95% confidence intervals)), mean ± standard deviation or median (interquartile range). For incompletely reported items, number of informative patients is given in italics. *MCD*, minimal-change disease; *MesPGN*, mesangio-proliferative glomerulonephritis; *FSGS*, focal segmental glomerulosclerosis; *eGFR*, estimated glomerular filtration rate; *CKD*, chronic kidney disease, *CKD stage 5*, kidney failure, and/or start of kidney replacement therapy

	Total	Non-Genetic	Genetic	Unknown
	*N* = 95	*N* = 45	*N* = 39	*N* = 11
**Characteristics at disease onset**				
***Age (years)***	6.1 ± 5.6	6.7 ± 5.7	4.9 ± 5.2	7.8 ± 6.5
***Age groups***				
3 mo to < 1 yr	22 (23 (15; 32) %)	9 (20 (8; 32) %)	11 (28 (14; 42) %)	2 (18 (0; 41) %)
1 to < 6 yrs	32 (34 (24; 43) %)	13 (29 (16; 42) %)	16 (41 (26; 56) %)	3 (27 (1; 54) %)
6 to < 12 yrs	22 (23 (15; 32) %)	13 (29 (16; 42) %)	7 (18 (6; 30) %)	2 (18.(0; 41%)
≥ 12 yrs	19 (20 (12; 28) %)	10 (22 (10; 34) %)	5 (13 (2; 23) %)	4 (36 (8; 65) %)
***Serum albumin (g/l)***	31.5 ± 11.7	32.1 ± 10.9	32.1 ± 12.5	27.5 ± 13.1
***Proteinuria***	*82*	*38*	*33*	*11*
Nephrotic range	67 (82 (73; 90) %)	31 (82 (69; 94) %)	27 (82 (69; 95) %)	9 (82 (59; 100) %)
Non-nephrotic range	13 (16 (8; 24) %)	7 (18 (6; 31) %)	4 (12 (1; 23) %)	2 (18 (0; 41) %)
No proteinuria	2 (2 (0; 6) %)	0	2 (6 (0; 14) %)	0
***Edema***				
Severe	5 (5 (1; 10) %)	4 (9 (1; 17) %)	1 (3 (0; 8) %)	0
Moderate	13 (14 (7; 20) %)	5 (11 (2; 20) %)	7 (18 (6; 30) %)	1 (9 (0; 26) %)
Mild	17 (18 (10; 26) %)	11 (24 (12; 37) %)	4 (10 (1; 20) %)	2 (18 (0; 41) %)
None	60 (63 (54; 73) %)	25 (56 (41; 70) %)	27 (69 (55; 84) %)	8 (73 (46; 99) %)
***Kidney function***	*76*	*37*	*31*	*8*
***eGFR***(ml/min*1.73m^2^/d)	97.8 (68.9; 136.5)	97.3 (68.3; 144.6)	103.8 (69.0; 138.4)	89.6 (72.5; 107.8)
***CKD Stage***	*76*	*37*	*31*	*8*
CKD 1	43 (57 (46; 68) %)	20 (54 (38; 70) %)	19 (61 (44; 78) %)	4 (50 (15; 85) %)
CKD 2	19 (25 (15; 35) %)	11 (30 (15; 44) %)	5 (16 (3; 29) %)	3 (38 (4; 71) %)
CKD 3	12 (16 (8; 24) %)	5 (14 (3; 25) %)	6 (19 (6; 33) %)	1 (13 (0; 35) %)
CKD 4	2 (3 (0; 6) %)	1 (3 (0; 8) %)	1 (3 (0; 9) %)	0
***Hypertension***	13 (14 (7; 21) %)	7 (16 (5; 26) %)	3 (8 (0; 16) %)	3 (27 (10; 54) %)
***Hematuria***	26 (28 (19; 37) %)	11 (24 (12; 37) %)	11 (28 (14; 42) %)	4 (40 (11; 69) %)
**Histopathological diagnosis**	*67*	*32*	*26*	*9*
MCD	9 (13 (5; 21) %)	4 (13 (1; 25) %)	3 (12 (0; 24) %)	2 (22 (0; 49) %)
MesPGN	8 (12 (4; 20) %)	4 (13 (1; 25) %)	4 (15 (2; 29) %)	0 (0%)
FSGS	40 (60 (48; 72) %)	20 (63 (46; 80) %)	17 (65 (47; 84) %)	3 (33 (3; 64) %)
Other	10 (15 (6; 24) %)	4 (13 (1; 25) %)	2 (8 (0; 18) %)	4 (44 (12; 77) %)
**Family history**	*75*	*33*	*31*	*11*
Positive	40 (53 (42; 65) %)	13 (39 (23; 56) %)	19 (61 (44; 78) %)	8 (73 (46; 99) %)
Negative	35 (47 (35; 58) %)	20 (61 (44; 77) %)	12 (39 (22; 56) %)	3 (27 (1; 54) %)
**Follow-up information**				
***Duration of observation (years)***	3.5 (1.7; 7.2)	3.3 (2.3; 6.1)	4.8 (1.4; 8.4)	2.5 (1.0; 5.1)
***Kidney failure during observation***				
No. of patients	18 (19 (11; 27) %)	2 (4 (0; 10) %)	13 (33 (19; 48) %)	2 (18 (0; 41) %)
Time to kidney failure (years)	2.3 (1.3; 4.1)	2.1 (1.7; 3.0)	3.2 (1.3; 9.1)	1.4 (0.9; 1.9)

More than 80% of children initially presented with CKD stages 1–2. Thirteen (33 (19; 48) %) children with genetic SRNS developed kidney failure during the observation period within 3.2 (1.3; 9.1) years of follow-up, whereas only 2 of 45 (4.4 (0; 10) %) children with non-genetic disease, thereof one child presenting with CKD3, progressed to kidney failure within 0.7 and 2.4 years.

The distribution of histopathological diagnoses was comparable in the subgroups, with a predominance of focal-segmental glomerulosclerosis (Table 1). The distribution of causative genetic defects is provided in Supplementary Table S-1.

### Treatment with RAAS antagonists

RAAS antagonist therapy was applied in all but 3 children (96.8 (93; 100) %). Treatment was started at 1.8 (0.2; 7.2) months after disease onset and was continued for 89 (61; 99)% of the observation time. The most frequently used ACE inhibitors (ACEi) were enalapril and ramipril. Angiotensin receptor blockers (ARB) were used rarely (3.3 (0; 7) %) as initial RAAS medication but were frequently administered in addition to ACEi or treatment was switched from ACEi to ARB. In total, 50 (34; 66) % of children with genetic disease and 26 (13; 39) % of those with non-genetic disease received dual RAAS blockade during the disease course. The average RAAS dosage during the observation period was 48.9 (27.3; 85.5) % of the maximal approved dosage (Table 2).

**Table 2:** **Treatment characteristics of the non-IIS study cohort. **Data are given as N (% (normal-approximated 95% confidence intervals)) or median (interquartile range). For incompletely reported items, number of informative patients is given in italics. *IIS*, intensified immunosuppressive treatment; *non-IIS*, children not treated with intensified immunosuppressive therapy; *RAAS*, renin–angiotensin–aldosterone system antagonist; *ACEi*, angiotensin converting enzyme-inhibitor; *ARB*, angiotensin-receptor-blocker

	Total Non-IIS	Non-Genetic	Genetic	Unknown
	*N* = 95	*N* = 45	*N* = 39	*N* = 11
**Type of treatment**				
RAAS	41 (43 (33; 53) %)	18 (40 (26; 54) %)	20 (51 (36; 67) %)	3 (27 (1; 54) %)
Steroids + RAAS	51 (54 (44; 64) %)	24 (53 (39; 68) %)	19 (49 (33; 64) %)	8 (73 (46; 99) %)
Steroids	3 (3.(0; 7) %)	3 (7 (0; 14) %)	0	0
**Treatment with oral steroids**				
No. of patients (%)	54 (57 (47; 67) %)	27 (60 (46; 74) %)	19 (49 (33; 64) %)	8 (73 (46; 99) %)
Total duration of oral steroids (months)	7.0 (3.0; 19.3)	6.7 (3.0; 15.7)	7.3 (2.7; 20.0)	7.1 (3.6; 37.5)
*1st prednisone treatment episode*				
Duration (months)	*34*	*18*	*10*	*6*
Prednisone daily	1.2 (1.0; 1.6)	1.3 (1.1; 1.6)	1.2 (1.1; 1.6)	1.0 (1.0; 1.1)
Prednisone a.d	1.4 (1.0; 4.3)	1.4 (1.0; 3.0)	3.1 (1.1; 6.3)	1.0 (0.3; 1.9)
**Treatment with RAAS**				
No. of patients (%)	92 (97 (93; 100) %)	42 (93 (86; 100) %)	39 (100%)	11 (100%)
Time from 1st man. to RAAS start (months)	1.8 (0.2; 7.2)	1.1 (0.1; 3.9)	2.3 (0.8; 14.5)	0.5 (0.1; 2.1)
**Type of initial RAAS treatment**				
ACEi	85 (92 (87; 98) %)	38 (91 (82; 99) %)	36 (95 (88; 100) %)	11 (100%)
ARB	3 (3 (0; 7) %)	1 (3 (0; 9) %)	2 (0; 12) %)	0
ACEi + ARB	4 (4 (0; 8) %)	3 (7 (0; 15) %)	1 (0; 8) %)	0
Combined ACEi + ARB during course of treatment	31 (34 (24; 43) %)	11 (26 (13; 39) %)	18 (50 (34; 66) %)	2 (18 (0; 41) %)
**RAAS dosage**				
Starting RAAS* doses (mg/kg/day)				
Enalapril (*n* = 57)	0.21 (0.13; 0.36)	0.20 (0.11; 0.40)	0.20 (0.13; 0.29)	0.30 (0.16; 0.40)
Ramipril (*n* = 32)	0.10 (0.06; 0.15)	0.08 (0.05; 0.14)	0.12 (0.08; 0.17)	-
Losartan (*n* = 32)	0.76 (0.54; 1.17)	1.06 (0.56; 1.61)	0.74 (0.53; 1.15)	0.79 (0.49; 1.09)
Maintenance RAAS* doses (mg/kg/day)				
Enalapril (*n* = 57)	0.21 (0.14; 0.34)	0.19 (0.12; 0.41)	0.22 (0.12; 0.32)	0.23 (0.15; 0.40)
Ramipril (*n* = 32)	0.11 (0.08; 0.16)	0.09 (0.05; 0.13)	0.14 (0.08; 0.22)	-
Losartan (*n* = 32)	0.75 (0.61; 1.35)	0.76 (0.67; 1.60)	0.70 (0.52; 1.23)	1.30 (0.59; 2.00)
% of max. approved maintenance dosage*				
Enalapril (max. 0.6 mg/kg/d)	34.5 (22.6; 56.8)	31.2 (20.2; 69.0)	36.6 (20.4; 52.7)	38.2 (25.5; 66.7)
Ramipril (max. 0.2 mg/kg/d)	54.7 (38.9; 81.6)	46.8 (23.6; 66.2)	69.4 (42.2; 111.2)	-
Losartan (max. 1.4 mg/kg/d)	53.8 (43.3; 96.2)	54.5 (48.2; 114.1)	49.7 (37.4; 88.0)	92.6 (42.1; 143.1)
*% of RAAS treatment during observation period*				
0–25%	10 (11 (5; 17)%)	4 (10 (1; 18) %)	4 (10 (1; 20) %)	2 (18 (0; 41) %)
> 25–50%	10 (11 (5; 17) %)	7 (17 (6; 28) %)	3 (8 (0; 16) %)	0
> 50–75%	11 (12 (6; 19) %)	4 (10 (1; 18) %)	6 (15 (4; 27) %)	1 (9 (0; 26) %)
> 75–100%	61 (66 (57; 76) %)	27 (64 (50; 78) %)	26 (74 (61; 88) %)	8 (73 (46; 91) %)
*% of RAAS treatment during observation period*	88.5 (61.0; 99.0)	92.0 (59.0; 99.0)	85.0 (65.0; 98.0)	92.0 (61.0; 91.0)
*Total RAAS exposure during treatment period (% max. approved doses)*	48.9 (27.3; 85.5)	41.6 (19.2; 85.0)	56.8 (38.1; 90.6)	47.6 (25.5; 66.7)

^*^Most frequently administered drugs

### Probability to achieve remission

**Fig. 2:**
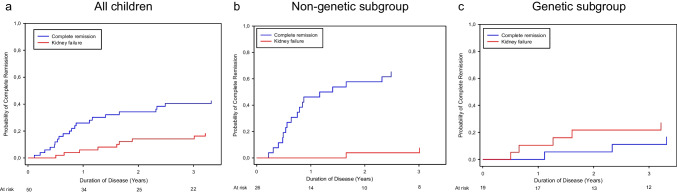
Cumulative probability of achieving complete remission in children with SRNS and nephrotic range proteinuria who were not treated with IIS. Competing risk analyses were performed evaluating the probability of achieving complete remission vs. kidney failure within 3 years after disease onset

The probability to achieve complete remission of proteinuria among all children presenting with nephrotic-range proteinuria (*n* = 67) was 12% (*n* = 6) at 6 months, 26% (*n* = 17) at 1 year, 34% (*n* = 23) at 2 years, and 40% (*n* = 28) at 3 years after disease onset (Fig. 2a).

**Fig. 3:**
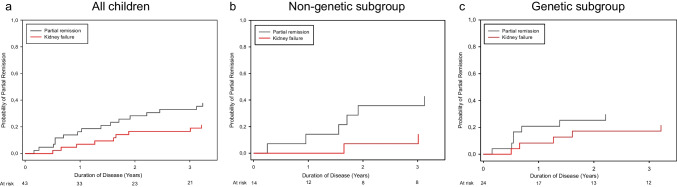
Cumulative probability of achieving partial remission as best response in children with SRNS and nephrotic range proteinuria who were not treated with IIS. Competing risk analyses were performed evaluating the probability of achieving partial remission as best response vs. kidney failure within 3 years after disease onset

Patients who did not achieve complete remission were evaluated for their likelihood to achieve partial remission. The cumulative probability of partial remission was 4.7% (*n* = 3) at 6 months, 16.3% (*n* = 11) at 1 year, 28.2% (*n* = 19) at 2 years, and 33% (*n* = 33) at 3 years after disease onset (Fig. 3a).

Stratification for genetic status showed distinct differences in remission rates between patients with confirmed genetic disease and those with apparently non-genetic SRNS.

In the children with non-genetic SRNS, complete remission was observed in 19.2% at 6 months, 46.2% at 1 year, 57.7% at 2 years, and 65.4% at 3 years after disease onset (Fig. 2b). Two patients progressed to kidney failure. Among the children without available genetic information, one out of five (diagnosed with infantile nephrotic syndrome) achieved transient complete remission.

Eighteen of 31 (58 (41; 75) %) children with non-genetic SRNS and initial nephrotic-range proteinuria achieved complete resolution of proteinuria without IIS treatment—8 (44 (21; 67) %) of those within 1 year of disease onset. Remission persisted in 11 of the 18 (61 (39; 84) %) children throughout the remaining observation period of 3.1 (2.6; 4.9) years.

Two children with familial SRNS relapsed with nephrotic-range proteinuria after a short remission period of 0.4 and 0.6 years and subsequently progressed from CKD 2 to CKD 3–4. Another two children developed non-nephrotic range proteinuria during further follow-up. In three children, lack of documentation after attainment of complete remission precluded assessment of the further course (Supplementary Table S-3).

Children with non-genetic SRNS and complete resolution of proteinuria first presented at a mean age of 4.3 (1.3; 10.2) years with a mean serum albumin of 2.9 (1.9; 3.6) g/dl and eGFR of 122 (79; 156) ml/min*1.73 m^2^). Initial steroid treatment consisted of daily oral prednisone at 63 (42; 107) mg/m^2^/d for 6 (5; 7) weeks and alternate daily prednisone for 7 (6; 13) weeks. Oral steroids were continued for an average of 6.4 (3.5; 11.5) months. Complete resolution of proteinuria occurred while on oral steroids (after 6 (4.5; 7.2) months of treatment) in 8 and off steroids in 10 children (Supplementary Table S-3).

RAAS antagonist therapy was started soon after SRNS diagnosis and was administered for 92 (41; 98) % of the observation period. Thirteen children received ACEi monotherapy and five dual RAAS blockade. Average RAAS exposure was 36 (19; 88) % of the maximal approved dose (Supplementary Table S-3).

Of those children with non-genetic SRNS who did not achieve complete remission, seven developed partial remission on RAAS antagonist therapy (Fig. 3b); partial remission was sustained in 5 of these under ongoing RAAS blockade (Supplementary Table S-3).

**Fig. 4:**
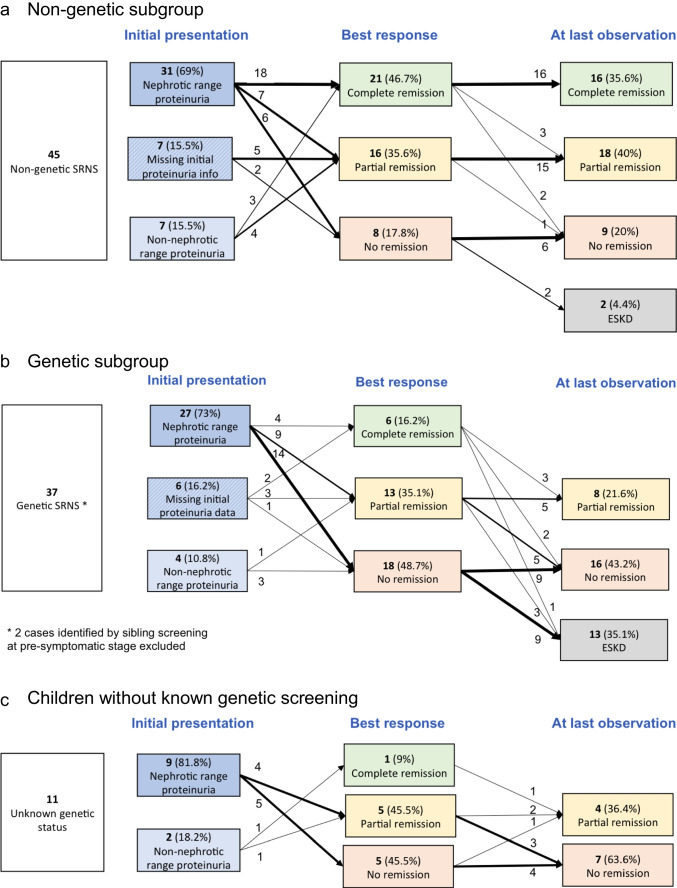
Outcome of all 95 children with non-IIS treatment: best response and at last observation with proteinuria measurement

The outcome of the children with non-genetic SRNS and initial non-nephrotic range proteinuria and missing initial proteinuria data, who were not included in the competing risk analysis, is shown in Fig. 4a. Three of 7 children with initial non-nephrotic range proteinuria achieved complete remission as well, and 4 remained with non-nephrotic range proteinuria during further follow-up. Furthermore, 5 of 7 children with missing initial proteinuria information showed partial remission (Fig. 4a).

In total, 21 of 45 (47 (32; 62%) children with non-genetic SRNS achieved complete remission, and another 16 (36 (22; 50) %) children achieved partial remission as best response. Sixteen of 45 children (36 (22; 50) %) showed sustained complete remission, and 18 (40 (26; 54) %) sustained partial remission at last proteinuria assessment (Fig. 4a).

In the children with genetic SRNS, remission was a very rare event that occurred only transiently (Figs. 2c and 3c, Supplementary Tables S-1 and S-2). Among 27 children with genetic SRNS presenting with nephrotic-range proteinuria, 2 children (one each with a heterozygous truncating *WT1* variant and *COL4A4* splice-site pathogenic variant) remitted completely for 6 months while on ACE inhibition. Two siblings with *PLCE1* biallelic truncating variants developed complete remission 3.3 and 8.3 years after disease onset, again while on ACE inhibition, for a period of 0.8 and 4.5 years (Supplementary Table S-2).

Nine children with genetic SRNS transiently achieved partial proteinuria remission for a duration of 1.4 (0.9; 2.6) years; all were on RAAS antagonist therapy and 7 on dual RAAS blockade. Relative RAAS exposure was 66 (55; 79) % of the approved dosage. At last observation, four of the 9 children with partial remission again showed persistent nephrotic-range proteinuria and another two had progressed to kidney failure.

Three of four children with non-nephrotic-range proteinuria at disease onset, not included in the competing risk analysis, later progressed to nephrotic-range proteinuria (Fig. 4b), whereas one child with *NUP93* podocytopathy showed stable mild proteinuria on ACEi within 3.6 years of follow-up. Two of six children with missing initial proteinuria information had a documented transient complete remission, and 3 had transient partial remission (Fig. 4b).

In all genetic SRNS patients, any episodes of proteinuria resolution were transient. At last observation, 16 (43 (27; 59) %) children showed nephrotic-range proteinuria and 13 (35 (20; 50%) had progressed to kidney failure (Fig. 4b). The outcome of 11 children with unknown genetic status is shown in Fig. 4c.

### Factors predicting complete remission

Univariate and bivariate Cox regression analyses were performed to identify factors predicting complete remission. Non-genetic etiology of SRNS and a higher relative RAAS antagonist exposure were independently associated with the probability of achieving complete remission, whereas no associations were found for age, serum albumin, eGFR, and histopathology at disease onset (Table 3).

**Table 3:** **Predictive factors for attainment of complete remission**. Univariate and bivariate Cox regression analysis was performed in order to identify predictive factors associated with complete remission

	Univariate			Bivariate		
Variable	HR	95%CI	*p*	HR	95%CI	*p*
**Characteristics at disease onset**						
*Age (years)*	1.04	0.96, 1.12	0.334		-	
eGFR (ml/min*1.73m^*2*^*/d)*	1.00	0.99, 1.02	0.731		-	
*Proteinuria* (ref = non-nephrotic range)						
Nephrotic range	1.53	0.21, 11.4	0.679		-	
*Serum albumin (g/l)*	1.02	0.99, 1.05	0.292		-	
**Cause of disease **(ref = non-genetic)						
Genetic	**0.17**	**0.06, 0.51**	**0.002**	**0.19**	**0.06, 0.68**	**0.011**
**Histopathology** (ref = MCD)						
All other	2.71	0.73, 10.02	0.135		-	
**RAAS Treatment**						
*RAAS treatment during observation period*	0.99	0.98, 1.01	0.313		-	
T*otal RAA*S e*xposure* (*per 10% of approved RAAS dose)*	**0.89**	**0.80, 0.99**	**0.032**	0.93	0.84, 1.02	0.129

## Discussion

Among 1041 children documented in the largest global SRNS database for this rare disease, 95 individuals never received any IIS treatment after primary steroid resistance was diagnosed. The most common reason for not initiating CNI therapy or other IIS was the early establishment of a genetic diagnosis or a high likelihood of genetic disease as evidenced by familial occurrence or associated extrarenal organ manifestations. However, in approximately half of the cases, there was no evidence or suspicion of a genetic disease origin; in these patients, IIS was withheld mainly due to a relatively mild phenotype or regressing proteinuria while on continued oral steroid therapy and/or RAAS inhibition.

This unique SRNS cohort in whom IIS standard of care was not administered allowed us to explore the natural history of SRNS, and genetic screening findings permitted us to compare the outcomes of genetic and apparently non-genetic forms of this heterogeneous disease. The specific outcomes of interest were, on the one hand, kidney failure-free survival and, on the other hand, the cumulative incidence of complete or partial disease remission. While solid estimates of the rates of kidney failure and proteinuria remission in response to IIS and in genetic disease have been established [6, 7, 9, 24], information on the disease evolution in SRNS patients undergoing only antiproteinuric RAAS inhibition is limited to a few case series [25–28]. Since the probabilities of entering remission or kidney failure are interrelated, we chose a competing risk approach to simultaneously monitor the likelihoods of the two events [29].

Among children with confirmed genetic disease 33% progressed to kidney failure within 3 years, in keeping with previous findings in the PodoNet and other cohorts where more than 50% patients with hereditary podocytopathies progressed to kidney failure within 5 years and more than 70% within 8–10 years, respectively [7, 9, 24]. In the patients with non-genetic SRNS, the 3-year incidence of kidney failure was very low, with only two patients progressing to kidney failure within the observation period. The very good short- to medium-term kidney survival observed in this group was comparable with that observed in fully CNI-responsive SRNS, the patient group with the best long-term outcomes in the PodoNet cohort [9]. It thus appears that at least within a 3-year perspective, the wait-and-see approach adopted in this pre-selected patient group with relatively mild initial disease activity did not lead to a rapid loss of kidney function (Supplementary Table S-4).

Even more remarkable insights were made regarding the proteinuria remission endpoint. In a subset of the genetic SRNS patients, proteinuria decreased in association with RAAS inhibition, formally leading to partial remission in 33% of patients. However, sustained complete remission was never observed, although anecdotally reported for short periods of time in very few patients [30, 31]. By contrast, in the non-genetic SRNS cases, complete remission was documented at a steadily increasing cumulative incidence throughout the first two years after disease onset, with 50% of patients reaching complete remission at 18 months. For comparison, 30% of the children in the PodoNet cohort who were exposed to CNI achieved complete remission within 12 months [9]. In addition to the 18 out of 31 children with non-genetic SRNS and initial nephrotic-range proteinuria who achieved complete remission, another 7 achieved partial remission, leaving only six, i.e., less than 20% of children with persistent nephrotic-range proteinuria during follow-up. The same remission rates were observed when children with initial non-nephrotic-range proteinuria and missing initial proteinuria information were included (21/45 complete remission, 16/45 partial remission).

The favorable outcome observed in patients with non-genetic SRNS and milder clinical phenotype not treated with IIS appears surprising, but is not inconsistent with previous literature. Twelve small case series altogether observed complete proteinuria remission in 20 out of 99 children with SRNS/FSGS who received RAAS antagonist therapy only [20, 21, 32–41]. Some of the children had previously demonstrated non-responsiveness to IIS. Given the fact that genetic cases were not excluded in most of these studies, the likelihood of complete remission in non-genetic SRNS was probably underestimated.

Our findings raise several questions regarding the cause and the clinical consequences of the high remission rate in the non-genetic cases with milder clinical phenotype in the absence of IIS. One might argue that some of the cases of early remission might reflect late responsiveness to oral steroid treatment. This is rather unlikely since all complete remissions occurred more than 8 weeks after initial disease manifestation, i.e., at a time when daily high-dose steroid therapy had been discontinued. In 10 patients, remission occurred after discontinuation of any oral steroid therapy. The high remission rates also could not be explained by progressive CKD as all complete remissions occurred in CKD stage 1 to 2. All patients received RAAS inhibitor therapy, which has been demonstrated both in retrospective case series [20, 21, 32–41] and controlled trials [42, 43] to reduce proteinuria by 35 to 80%. The 20 previously reported SRNS cases with complete remission of nephrotic range proteinuria on RAAS blockade were described in uncontrolled case series [20, 21, 32–41]; hence, spontaneous remission could not be ruled out. Notably, we did not find any relationship between the relative RAAS antagonist dosage and the likelihood of complete remission. Median exposure was only 42% of the approved maximal drug dosage, possibly implicating that dose escalation was not required because proteinuria was already regressing. Also, higher RAAS antagonist exposure was noted in the patients with genetic SRNS, where sustained complete remission did not occur, than in the non-genetic group. Taken together, while RAAS antagonist therapy probably contributed to proteinuria reduction and may explain some of the partial remission cases, we found little evidence for a pharmacological cause of the widespread complete proteinuria remission in patients with non-genetic SRNS. This leaves the possibility that this condition, at least when presenting with a mild to moderate phenotype, may frequently resolve spontaneously. Spontaneous remission is a well-established outcome in other glomerular diseases presenting with nephrotic syndrome, including post-infectious glomerulonephritis, IgA nephropathy/vasculitis [44, 45], membranous nephropathy [23], and dense-deposit disease [46, 47].

Although our study represents the largest longitudinal case collection of primary SRNS cases without IIS, the analysis still faced limitations. The very large size of the PodoNet cohort and the comprehensive and long-term data collection are major strengths of this international study. On the other hand, analyses of the PodoNet cohort are limited by potential selection bias of cases enrolled into this voluntary registry study and by incompleteness of reporting. We queried the contributing centers to confirm the non-IIS treatment approach, obtain any missing information and update patient follow-up. Genetic screening information was obtained in 84 of the 95 included children (88.4 (82; 95) %). Information on viral induced etiologies was available in only one-third of the cohort. The most important limitation of the study presented here was the obvious pre-selection of cases for withholding IIS treatment according to their mostly milder clinical disease severity of SRNS. Hence, our study represents a hypothesis-generating analysis but in no way replaces a prospective randomized trial comparing IIS and non-IIS approaches in pediatric SRNS. Finally, the size of the non-IIS cohort was insufficient to unequivocally identify predictors of remission other than genetic status.

Notwithstanding these limitations, our findings have important potential implications for the interpretation of previous IIS study results, derived best practice recommendations and future clinical trials. First, we confirmed the paramount importance of genetic screening for risk stratification. Secondly, the observed potential at least of the milder non-genetic SRNS cases to remit spontaneously highlights the possibility that the IIS responder rates reported in previous trials and observational studies devoid of untreated control arms were overestimations due to a substantial rate of spontaneous recoveries, at least in patients with mild to moderate clinical phenotypes. Thirdly, future trials in non-genetic SRNS should include risk stratification procedures and consider the inclusion of untreated control arms. Finally, it might be appropriate for future clinical practice recommendations to adopt risk-adapted, individualized management strategies and to apply a wait-and-see approach in children with mild to moderate presentation, particularly in those with spontaneously regressing proteinuria.

## Supplementary Information

Below is the link to the electronic supplementary material.

Graphical Abstract (PPTX 206 KB)

Supplementary file2 (PDF 262 KB)
