# Supplementary material for: Outcomes of steroid-resistant nephrotic syndrome in children not treated with intensified immunosuppression
Source: Pediatr Nephrol. 2022 Oct 31;38(5):1499–511. doi: 10.1007/s00467-022-05762-4 (PMC10060323; doi:10.1007/s00467-022-05762-4)
Supplement: Supplementary file 1 — Graphical Abstract (PPTX 206 KB) [file 467_2022_5762_MOESM1_ESM.pptx]

## Slide 1
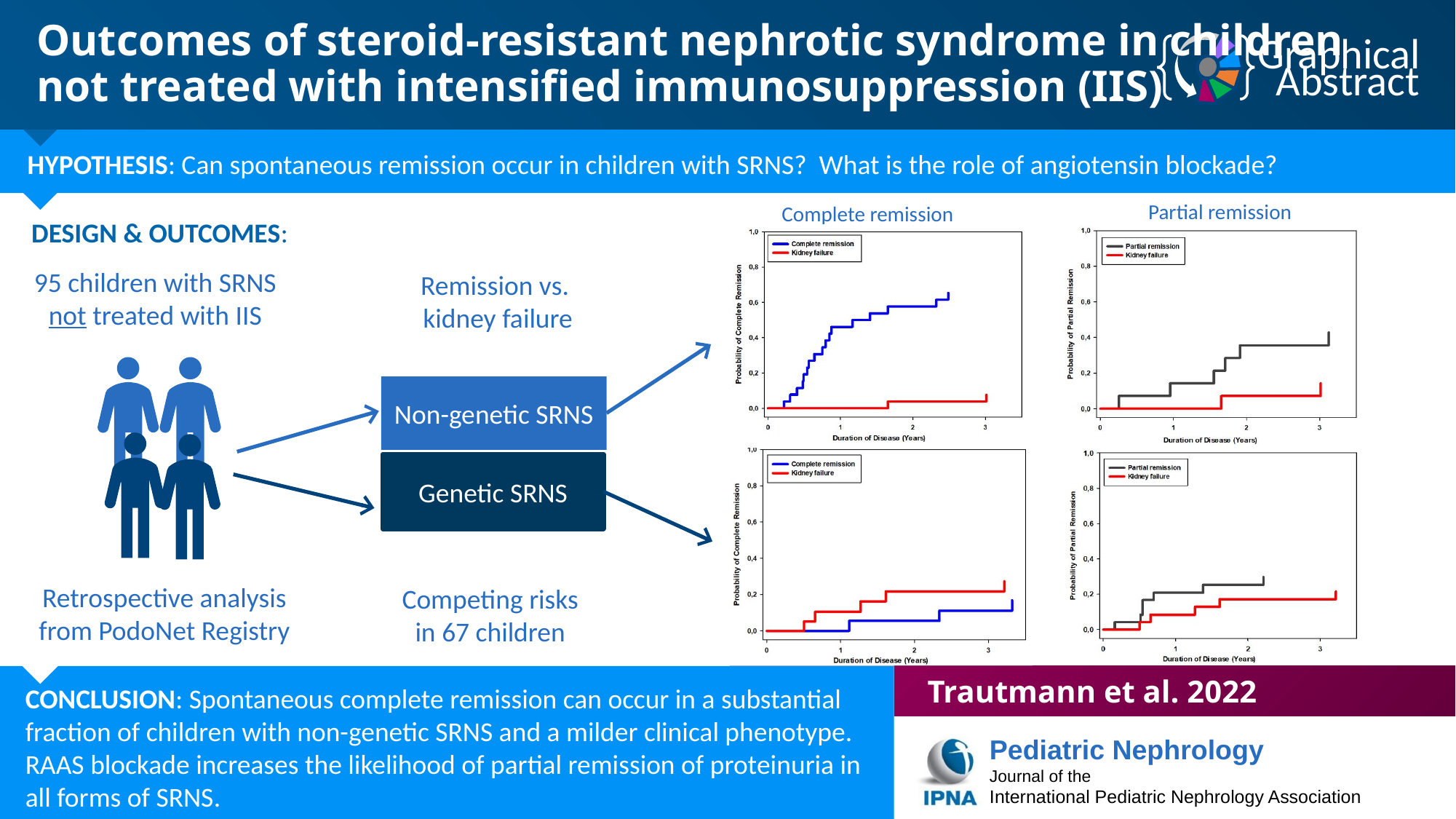

Outcomes of steroid-resistant nephrotic syndrome in children
not treated with intensified immunosuppression (IIS)
HYPOTHESIS: Can spontaneous remission occur in children with SRNS? What is the role of angiotensin blockade?
Partial remission
Complete remission
DESIGN & OUTCOMES:
95 children with SRNS
not treated with IIS
Remission vs.
kidney failure
Non-genetic SRNS
Genetic SRNS
Retrospective analysis from PodoNet Registry
Competing risks
in 67 children
Trautmann et al. 2022
CONCLUSION: Spontaneous complete remission can occur in a substantial fraction of children with non-genetic SRNS and a milder clinical phenotype.
RAAS blockade increases the likelihood of partial remission of proteinuria in all forms of SRNS.
