# Supplementary material for: Outcomes of steroid-resistant nephrotic syndrome in children not treated with intensified immunosuppression
Source: Pediatr Nephrol. 2022 Oct 31;38(5):1499–511. doi: 10.1007/s00467-022-05762-4 (PMC10060323; doi:10.1007/s00467-022-05762-4)
Supplement: Supplementary file 2 — Supplementary file2 (PDF 262 KB) [file 467_2022_5762_MOESM2_ESM.pdf]

Outcomes of steroid-resistant nephrotic syndrome in children not treated with intensified immunosuppression

Agnes Trautmann[1], Svenja Seide[2], Beata S. Lipska-Ziętkiewicz[3], Fatih Ozaltin[4], Maria Szczepanska[5], Marta Azocar[6], Augustina Jankauskiene[7], Alexandra Zurowska[8], Salim Caliskan[9], Bassam Saeed[10], William Morello[11], Francesco Emma[12], Mieczyslaw Litwin[13], Alexey Tsygin[14], Svitlana Fomina[15], Anna Wasilewska[16], Anette Melk[17], Elisa Benetti[18], Jutta Gellermann[19], Natasa Stajic[20], Marcin Tkaczyk[21], Sergey Baiko[22], Larisa Prikhodina[23], Dagmar Csaicsich[24], Anna Medynska[25], Regina Krisam[2], Heike Breitschwerdt[2], Franz Schaefer[1] for the PodoNet Consortium.

[1] Division of Pediatric Nephrology, University Center for Pediatrics and Adolescent Medicine, Heidelberg, Germany
[2] Institute of Medical Biometry, University of Heidelberg, Germany
[3] Rare Diseases Centre and Clinical Genetics Unit, Department of Biology and Medical Genetics, Medical University of Gdansk, Gdansk, Poland
[4] Department of Pediatric Nephrology, Nephrogenetics Laboratory and Center for Biobanking and Genomics, Hacettepe University, Ankara, Turkey
[5] Department of Pediatrics, School of Medicine with the Division of Dentistry, Zabrze, Poland
[6] Pediatric Nephrology, Hospital Luis Calvo Mackenna-Facultad de Medicina Universidad de Chile, Santiago de Chile
[7] Pediatric Center, Institute of Clinical medicine, Vilnius University, Lithuania
[8] Department of Pediatrics, Nephrology & Hypertension, Medical University of Gdansk, Gdansk, Poland
[9] Pediatric Nephrology Departement, Cerrahpaşa Medical Faculty, Istanbul University-Cerrahpasa, Turkey
[10] Farah Association for Child with Kidney Disease in Syria, Damascus, Syria
[11] Pediatric Nephrology, Dialysis and Transplant Unit, Fondazione IRCCS Ca'Granda, Ospedale Maggiore Policlinico, Milano, Italy
[12] Department of Pediatric Subspecialties, Nephrology and Dialysis Unit, Children's Hospital Bambino Gesù, IRCCS, Rome, Italy
[13] Department of Pediatric Nephrology, The Children's Memorial Health Center, Warsaw, Poland
[14] National Medical and Research Center for Children's Health, Moscow, Russia
[15] Department of Pediatric Nephrology, State Institution "Institute of Nephrology of NAMS of Ukraine", Kyiv, Ukraine
[16] Department of Pediatric Nephrology, University Hospital, Bialystok, Poland
[17] Department of Pediatric Kidney, Liver and Metabolic Diseases, Hannover Medical School, Hannover, Germany
[18] Pediatric Nephrology, Dialysis and Transplant Unit, Department of Women's and Children's Health, Padua University Hospital, Padua, Italy
[19] Clinic for Pediatric Nephrology, Charite Hospital, Berlin, Germany
[20] Department of Pediatric Nephrology, Institute of Mother and Healthcare of Serbia, Belgrade, Serbia
[21] Pediatric Nephrology Division, Polish Mothers Memorial Hospital Research Institute, Lodz, Poland
[22] National Center for Pediatric Nephrology and Renal Replacement Therapy, Belarusian State Medical University, Minsk, Belarus
[23] Division of Inherited & Acquired Kidney Diseases, Research & Clinical Institute for Pediatrics, Pirogov Russian National Research Medical University, Moscow, Russia
[24] Department of Pediatrics, Medical University Vienna, Vienna, Austria
[25] Department of Pediatric Nephrology, Wroclaw Medical University, Wroclaw, Poland

SUPPLEMENTAL MATERIAL:

CONTENT:

Table S-1: Molecular diagnosis in the 39 patients diagnosed with genetic SRNS.

Table S-2: Characteristics of children with genetic podocytopathies and reported transient complete or partial proteinuria normalization.

Table S-3: Characteristics and outcome of children with non-genetic SRNS and non-IIS treatment

Table S-4: Change of eGFR in children with non-genetic SRNS within the observation period of 3 years

Table S-1: Molecular diagnosis in the 39 patients diagnosed with genetic SRNS.

**Causative gene**	**Frequency N (%)**	**Transient/ sustained Complete rem.**	**Transient/ sustained Partial rem.**
NPHS2	10 (25.6)	-	1 / -
WT1	6 (15.4)	1 / -	2 / 1
PLCE1	5 (12.8)	2 / -	1 / 1
SMARCAL1	3 (7.7)	-	-
COL4A5	2 (5.6)	-	1 / -
COL4A4	2 (5.6)	1 / -	1 / -
CLCN5	2 (5.6)	-	-
NUP93	2 (5.6)	1 / -	-
NPHS1	1 (2.6)	-	-
INF2	1 (2.6)	-	1 / 1
COQ8B	1 (2.6)	-	1 / -
COQ6	1 (2.6)	1 / -	-
NUP205	1 (2.6)	-	-
Microdeletion at chrom. 11p	1 (2.6)	-	-
Microduplication at chrom. 6p	1 (2.6)	-	1 / -
**All**	**39**	**6 / -**	**9 / 3**

Table S-2: Characteristics of children with genetic podocytopathies and reported transient complete or partial proteinuria normalization.

Abbreviations: RAAS antagonist = renin-angiotensin aldosterone system antagonist, CKD = chronic kidney disease, Kidney failure = CKD stage 5 and/or start of kidney replacement therapy. Hom. – homozygote; Het. – heterozygote; Comp. Het. – compound heterozygote

Reference sequences: *COL4A4* (NM_000092.5); COL4A5(*NM_033380.3*); *COQ8B* (NM_024876.4); *INF2* (NM_022489.4); *NPHS2* (NM_014625.4); *PLCE1* (NM_016341.4); *WT1* (NM_024426.6)

Gene	Mutation	Age at disease onset (yrs)	S-albumin at disease onset (g/l)	Familial disease	Remission – time after disease onset (yrs)	Duration of remission (yrs)	RAAS Antagonist (± oral steroid)	RAAS Exposure (% of max. approved dose)	Follow-up (yrs)	Remission status at last observation	Status at last observation
**Nephrotic-range proteinuria -> Complete remission (Primary analysis)**											
WT1	c.1387C>T p.(Arg463Ter) het. (alias p.R390X, p.Arg458Ter)	0.9	39	no	1.1	0.5	Ramipril	65.1%	2.1	Partial rem.	CKD2
COL4A4	c.2968+2T>C hom.	8.4	45	yes	2.3	0.5	Enalapril	63.3%	4.8	No rem.	CKD1
PLCE1	c.5560C>T p.(Gln1854Ter) hom.	0.8	44	yes	3.3	0.8	Enalapril	37.2%	13.1	Partial rem.	CKD 1
PLCE1	c.5560C>T p.(Gln1854Ter) hom.	1.0	43	yes	8.3	4.5	Enalapril	13.3%	18.6	Partial rem.	CKD 1
**Nephrotic-range proteinuria -> Partial remission (Secondary analysis)**											
COQ8B	c.293T>G p.(Leu98Arg) hom.	13.5	35	yes	0.6	0.8	Enalapril, Losartan	97.2%	2.4	No rem.	Kidney failure
COL4A4	c.2968+2T>C hom..	10.1	37	yes	1.4	2.0	Enalapril, Losartan	65.7%	5.4	No rem.	CKD1
COL4A5	c.4750A>C p.(Ser1584Arg) het.	16.3	43	yes	0.6	0.8	Ramipril, Losartan	38.1%	1.4	No rem.	CKD1
INF2	c.652C>T p.(Arg218Trp) het.	16.7	50	yes	0.7	-	Ramipril	30.7%	0.7	Partial rem.	CKD1
NPHS2	c.506T>C p.(Leu169Pro) hom.	4.5	n.a.	yes	2.2	4.5	Enalapril, Losartan	78.8%	7.2	No rem.	CKD2
PLCE1	c.1477C>T p.(Arg493Ter); c.5891G>T p.(Arg1964Leu) comp. het.	1.4	n.a.	no	0.2	0.9	Enalapril	56.8%	1.1	Partial rem.	CKD1
WT1	c.1387C>T p.(Arg463Ter) het. (alias p.R390X, p.Arg458Ter)	18.5	40	no	0.5	7.5	Ramipril, Losartan	67.7%	9.0	Partial rem.	CKD2
WT1	c.1399C>T p.(Arg467Trp) het. alias (p.R394W, p.Arg462Trp)	1.3	23	unknown	3.0	1.8	Ramipril, Losartan	174.7%	6.1	No rem.	CKD1
arr[hg18]	6p25.3(92934-1172037)x1, 6p25.3p22.1(1172038-29167599)x3	0.7	24	no	8.4	0.9	Enalapril, Losartan	54.9%	9.3	Transplanted	Transplanted

Table S-3: Characteristics and outcome of children with non-genetic SRNS and non-IIS treatment.

Abbreviations: RAAS antagonist = renin-angiotensin aldosterone system antagonist, CKD = chronic kidney disease, Kidney failure = CKD stage 5 and/or start of kidney replacement therapy

Age at disease onset (yrs)	S-albumin at disease onset (g/l)	Kidney function at disease onset	Familial disease	Remission – time after disease onset (yrs)	Duration of remission (yrs)	Duration of steroid treatment (yrs)	RAAS Antagonist (± oral steroid)	RAAS Exposure (% of max. approved dose)	Follow-up (yrs)	Remission status at last observation	Status at last observation
**Nephrotic range proteinuria -> Complete remission**											
2.0	29	CKD I	yes	0.6	4.3	0.6	Enalapril	27.8	4.9	Complete rem.	CKD I
13.2	29	CKD II	no	0.5	2.1	2.5	Enalapril	20.9	2.6	Complete rem.	CKD I
13.8	35	CKD II	yes	0.5	0.6	1.5	Enalapril, Losartan	80.2	1.8	No remission	CKD IV
6.4	45	CKD I	Not defined	0.8	6.7	-	Enalapril, Losartan	46.1	10.1	Partial rem.	CKD II
16.0	44	CKD II	Not defined	1.2	1.5	-	Enalapril, Losartan	38.1	2.6	Complete rem.	CKD I
10.8	40	CKD III	Not defined	2.5	2.6	0.1	Enalapril	18.7	5.1	Complete rem.	CKD III
2.2	22	CKD I	no	0.2	2.5	0.3	-	-	2.7	Complete rem.	n.a.
1.1	14	n.a.	no	0.6	-	0.6	Enalapril	15.2	0.6	Complete rem.	CKD I
4.2	28	CKD I	no	0.9	-	0.3	Enalapril	8.6	0.9	Complete rem.	CKD I
10.3	18	CKD II	Not defined	1.7	1.7	0.5	Ramipril	36.3	3.3	Complete rem.	CKD I
0.5	36	CKD I	no	0.3	2.4	0.3	Ramipril	23.6	2.7	Complete rem.	CKD I
2.2	19	CKD III	no	0.8	2.3	0.8	Enalapril	90.4	3.1	Complete rem.	CKD I
0.3	19	CKD I	no	0.6	2.5	0.2	Enalapril	91.2	3.1	Complete rem.	CKD I
0.4	16	CKD I	no	3.3	2.3	0.2	Enalapril, Losartan	112.3	5.6	Complete rem.	CKD I
10.0	35	CKD II	yes	0.9	0.4	-	Ramipril	87.7	3.2	No remission	CKD III
1.0	19	CKD I	no	4.5	4.0	2.6	Enalapril	15.9	8.5	Complete rem.	CKD I

Age at disease onset (yrs)	S-albumin at disease onset (g/l)	Kidney function at disease onset	Familial disease	Remission – time after disease onset (yrs)	Duration of remission (yrs)	Duration of steroid treatment (yrs)	RAAS Antagonist (± oral steroid)	RAAS Exposure (% of max. approved dose)	Follow-up (yrs)	Remission status at last observation	Status at last observation
4.3	36	CKD I	no	0.4	-	0.4	Ramipril	18.5	0.4	Complete rem.	CKD I
8.4	39	CKD I	Not defined	1.4	3.3	1.1	Ramipril, Losartan	116.2	4.7	Partial rem.	CKD I
**Nephrotic-range proteinuria -> Partial remission**											
8.0	38	CKD III	yes	1.9	7.0	0.02	Enalapril, Losartan	50.2	10.2	No remission	CKD IV
7.3	48	CKD I	yes	1.6	2.0	-	Enalapril	n.a.	3.5	Partial rem.	CKD I
6.6	24	CKD I	no	1.0	5.7	6.6	Enalapril, Losartan	82.4	6.6	Partial rem.	CKD II
2.4	30	CKD II	Not defined	0.3	2.0	0.3	Enalapril, Losartan	283.1	2.3	Partial rem.	CKD I
6.1	42	n.a.	Not defined	3.1	5.8	-	Enalapril, Losartan	105.1	8.9	Partial rem.	CKD I
1.3	-	n.a.	Not defined	6.5	1.6	0.7	Ramipril	66.2	8.2	Partial rem.	CKD I
13.0	37	CKD II	yes	1.7	0.7	-	Ramipril	66.2	2.4	n.a.	CKD III
**Nephrotic-range proteinuria -> No remission**					**Time to Kidney failure (yrs)**						
0.4	44	CKD I	Not defined	-	-	-	Enalapril	18.0	5.4	No remission	CKD I
0.3	24	CKD I	yes	-	-	-	Captopril	15.8	0.9	No remission	CKD IV
19.8	40	CKD III	yes	-	2.4	-	Irbesartan	155.7	3.1	No remission	Kidney failure
5.8	7	CKD I	no	-	0.7	-	Enalapril	45.0	0.7	No remission	Kidney failure, deceased
0.3	28	CKD II	no	-	-	1.56	Enalapril	17.3	5.1	No remission	CKD II
0.5	18	CKD II	Not defined	-	-	0.02	Enalapril, Losartan	227.9	1.0	No remission	CKD I

Table S4: Change of eGFR in children with non-genetic SRNS (n=45) within the observation period of 3 years

Estimated glomerular filtration rate (eGFR) given as median (IQR) and mean ± SD; eGFR normalised to a maximum of 120 ml/min*1.73 m^2 to avoid potential bias from hyperfiltration phenomenon

**Change of eGFR (ml/min*1.73 m^2) after 1st manifestation**	**Baseline eGFR**	**6 months (n=43)**	**1 year (n=39)**	**2 years (n=35)**	**3 years (n=31)**	**Cumulative within 3 years**
**Median (IQR)**	98.2 (68.3; 120.0)	0 (0; 3.7)	0 (-0.3; 0.7)	0 (-0.4; 2.2)	0 (-3.5; 1.4)	0 (-9.0; 13.5)
**Mean (SD)**	92.0 ± 28.8	4.4 ± 23.2	-0.7 ± 7.8	0.4 ± 11.7	-1.0 ± 11.7	3.7 ± 29.9
